# Growth Hormone Increases BDNF and mTOR Expression in Specific Brain Regions after Photothrombotic Stroke in Mice

**DOI:** 10.1155/2022/9983042

**Published:** 2022-04-15

**Authors:** Sonia Sanchez-Bezanilla, Daniel J. Beard, Rebecca J. Hood, N. David Åberg, Patricia Crock, Frederick R. Walker, Michael Nilsson, Jörgen Isgaard, Lin Kooi Ong

**Affiliations:** ^1^School of Biomedical Sciences and Pharmacy and the Priority Research Centre for Stroke and Brain Injury, The University of Newcastle, NSW, Australia; ^2^Hunter Medical Research Institute, NSW, Australia; ^3^Institute of Medicine, The Sahlgrenska Academy, University of Gothenburg, Gothenburg, Sweden; ^4^Department of Acute Medicine and Geriatrics, Region Västra Götaland, Sahlgrenska University Hospital, Gothenburg, Sweden; ^5^Department of Paediatric Endocrinology and Diabetes, John Hunter Children's Hospital, NSW, Australia; ^6^NHMRC Centre of Research Excellence Stroke Rehabilitation and Brain Recovery, VIC, Australia; ^7^Centre for Rehab Innovations, The University of Newcastle, NSW, Australia; ^8^LKC School of Medicine, Nanyang Technological University, Singapore; ^9^Department of Specialist Medicine, Region Västra Götaland, Sahlgrenska University Hospital, Gothenburg, Sweden; ^10^School of Pharmacy, Monash University Malaysia, Selangor, Malaysia

## Abstract

**Aims:**

We have shown that growth hormone (GH) treatment poststroke increases neuroplasticity in peri-infarct areas and the hippocampus, improving motor and cognitive outcomes. We aimed to explore the mechanisms of GH treatment by investigating how GH modulates pathways known to induce neuroplasticity, focusing on association between brain-derived neurotrophic factor (BDNF) and mammalian target of rapamycin (mTOR) in the peri-infarct area, hippocampus, and thalamus.

**Methods:**

Recombinant human growth hormone (r-hGH) or saline was delivered (0.25 *μ*l/hr, 0.04 mg/day) to mice for 28 days, commencing 48 hours after photothrombotic stroke. Protein levels of pro-BDNF, total-mTOR, phosphorylated-mTOR, total-p70S6K, and phosporylated-p70S6K within the peri-infarct area, hippocampus, and thalamus were evaluated by western blotting at 30 days poststroke.

**Results:**

r-hGH treatment significantly increased pro-BDNF in peri-infarct area, hippocampus, and thalamus (*p* < 0.01). r-hGH treatment significantly increased expression levels of total-mTOR in the peri-infarct area and thalamus (*p* < 0.05). r-hGH treatment significantly increased expression of total-p70S6K in the hippocampus (*p* < 0.05).

**Conclusion:**

r-hGH increases pro-BDNF within the peri-infarct area and regions that are known to experience secondary neurodegeneration after stroke. Upregulation of total-mTOR protein expression in the peri-infarct and thalamus suggests that this might be a pathway that is involved in the neurorestorative effects previously reported in these animals and warrants further investigation. These findings suggest region-specific mechanisms of action of GH treatment and provide further understanding for how GH treatment promotes neurorestorative effects after stroke.

## 1. Introduction

Growth hormone (GH) treatment is emerging as a promising therapy in numerous neurological conditions including traumatic brain injury [1] and stroke [2–4]. In addition to its classical actions on growth and metabolism, when delivered therapeutically, GH has been linked to many neurorestorative effects within the CNS, including enhanced neuro-, vasculo-, and synaptogenesis, as well as the promotion of myelination [5, 6].

The therapeutic potential of GH after stroke has been considered in both preclinical [7, 8] and clinical studies [2–4]. In previous studies, our group has demonstrated that GH treatment promotes brain repair after experimental stroke [7, 9, 10]. Specifically, we demonstrated that GH treatment starting 48 hours poststroke for 28 days promotes the expression of several growth factors (including insulin-like growth factor-1 (IGF-1) and vascular endothelial growth factor (VEGF)), synaptic plasticity, and proliferation of neural progenitor cells in both the peri-infarct area and the hippocampus. These neuroregenerative effects were also associated with both cognitive and motor function improvement [7, 9, 10]. Therefore, it is worthwhile to understand the pleiotropic effects of GH on the brain, particularly at the signalling pathways related to neurorestoration.

GH treatment has been shown to increase expression of brain-derived neurotrophic factor (BDNF) and improve cognitive outcomes in traumatic brain injury [11]. Further, GH has been shown to increase BDNF in the retina and improve neuroregeneration following excitotoxic retinal injury [12]. BDNF is a neurotrophic growth factor that can maintain neuronal survival and plays an important role in synaptogenesis by binding to tropomyosin receptor kinase B (TrkB) receptors [13]. TrkB is known to signal through AKT leading to activation of mammalian target of rapamycin complex 1 (mTORC1) via phosphorylation at the Ser2448 [14]. mTORC1 is expressed in neurons and modulates key translational processes by direct or indirect activation of p70S6K via the phosphorylation at Thr389 [15]. mTORC1 has been shown to play a key role in neurodegeneration after stroke, with evidence showing that inhibition of mTORC1 with rapamycin during the acute phases of stroke is neuroprotective [16]. It should be noted that mTORC1 also plays a role in activity-dependent translation of proteins required for synaptic plasticity (e.g., *α*-amino-3-hydroxy-5-methyl-4-isoxazolepropionic acid (AMPA) receptor subunits), neurogenesis, and synapse formation [17].

However, there is still a gap in understanding how GH modulates BDNF and mTOR signalling in different brain regions, leading to brain recovery in ischemic stroke. GH treatment has shown great potential for neurorestoration at both the primary site of infarction and the hippocampus (an area that has been shown to suffer poststroke secondary neurodegeneration). The thalamus is another area of the brain that suffers secondary neurodegeneration [18–20]. We were interested in examining whether the mechanisms that promote neurorestoration in the peri-infarct area and hippocampus are the same in the thalamus. This study represents the extension of a previous study carried out by our group [9, 10]. The aim of this study was to determine whether treatment with GH alters BDNF levels in different regions of the brain poststroke and if this is associated with an alteration in mTOR signalling (total-mTOR (T-mTOR), phosphorylated-mTOR (P-mTOR), total-p70S6K (T-p70S6K), and phosphorylated-p70S6K (P-p70S6K)) as well as markers associated with neuro- and synaptogenesis in these brain regions. The purpose of this hypothesis-generating study was to explore how GH modulates pathways known to induce neuroplasticity, which could then be tested in subsequent studies.

## 2. Methods

### 2.1. Animals

All animal experiments were approved by the University of Newcastle Animal Care and Ethics Committee (A-2014-432) and undertaken in accordance with the ARRIVE guidelines [21]. This study represents an extension of a previous study [9, 10]. Therefore, the materials from the same mice cohort were used to obtain the data shown in this paper. This is in line with the aim to improve the ethical use of animals in testing according to the 3R principle [22].

### 2.2. Experimental Design

Briefly, C57BL/6 mice (male, 10 weeks old, *n* = 24) were obtained from the Animal Services Unit at the University of Newcastle, Australia. For day 0, mice were randomly allocated to photothrombotic occlusion or sham surgery (stroke, *n* = 18, and sham, *n* = 6). Previous studies have reported the beneficial effect of r-hGH treatment at 5-day postbrain injury [23, 24], as well as immediate treatment for 4 days, commencing at 10 days for a duration of 4 days [25], and at longer time frames of 2-6 weeks after stroke [8, 24]. As we wanted to explore mechanisms and details primarily from regenerative (long-term) effects of GH, we set out to determine the benefits of a 28-day long-term GH treatment poststroke. Therefore, for that reason, on day 2, stroke mice were further randomized to receive recombinant human GH (r-hGH) or saline at 1.4 mg/kg body weight per day subcutaneously via mini-osmotic pumps for 28 days, and sham mice just received saline (Sham+Saline, *n* = 6; Stroke+Saline, *n* = 8; and Stroke+r-hGH, *n* = 10). We administered r-hGH via osmotic minipumps to reduce stress-related daily intraperintoneal (IP) injections, as our previous work has shown that stress worsens stroke recovery [19]. Nevertheless, IP injections could be more physiological than continuous infusions of GH. Although similar in the direction of effect, IP injections indeed have been shown to cause more robust effects than IV infusions, peripherally [26] and in the brain [27].

Brains were collected at 30 days poststroke for western blotting. One mouse from the Stroke+r-hGH group had to be excluded due to no stroke.

### 2.3. Photothrombotic Stroke and r-hGH Treatment

Photothrombotic occlusion was performed as described previously [7, 9, 28]. Briefly, under isoflurane anesthesia (2%), the mouse skull was exposed via midline scalp incision. Mice received an intraperitoneal injection of rose bengal (200 *μ*l, 10 mg/ml solution in sterile saline, Sigma-Aldrich, USA) or 200 *μ*l of sterile saline (0.9% NaCl, Pfizer, Australia) for sham animals. After 8 min, the skull was illuminated for 15 min by a 4.5 mm diameter cold light source positioned above the left motor and somatosensory cortices (2.2 mm lateral to Bregma 0.0 mm).At 48 hours poststroke, a mini-osmotic pump (Model 2004, Alzet, USA) filled with 200 *μ*l of either r-hGH (somatropin 10 mg/1.5 ml, SciTropin A, SciGen, Australia) or sterile saline was inserted between the scapulae as previously described [7, 9, 10]. The pumps deliver 0.25 *μ*l/hour for 28 days (0.04 mg r-hGH/day).

### 2.4. Tissue Processing

**Figure 1:**
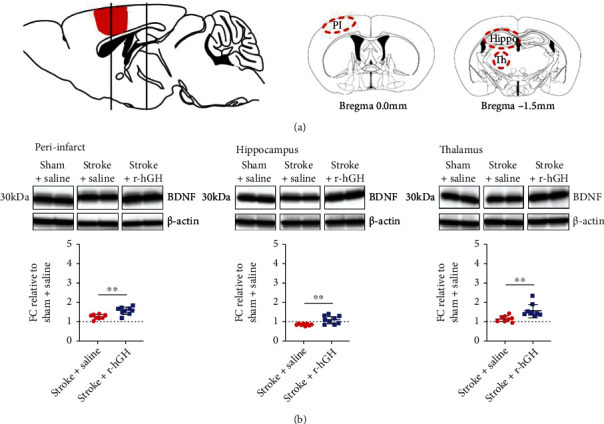
The effect of recombinant human growth factor (r-hGH) treatment poststroke on uncleaved immature form of brain derived neurotrophic factor (pro-BDNF, p-BDNF) expression. (a) Sagittal diagram shows the location of photothrombotic stroke induction (red area) at Bregma 0.0 mm. The black lines represent the location of tissue collection from the peri-infarct cortex at Bregma 0.0 mm as well as the hippocampus and thalamus at Bregma -1.5 mm. Coronal slices (mid and right panels) are also shown at 0.0 mm with peri-infarct region and -1.5 mm with hippocampus and thalamus regions. Red circles represent the area selected for western blot analyses. (b) Representative blots of pro-BDNF and *β*-actin in the top panels for peri-infarct, hippocampus, and thalamus. Loading controls were performed by loading equal amounts of total protein and also were normalized to *β*-actin. Levels were expressed as a fold change (FC) of mean ± SD for each group relative to the mean of the Sham+Saline group (dotted line), shown in the bottom panels for each location. We found a significant increase in pro-BDNF within the peri-infarct, hippocampus, and thalamus in stroked mice treated with r-hGH (blue squares) compared with saline (red circles). ∗∗*p* < 0.01, 2-tailed *t*-test.

Mice were anesthetized with sodium pentobarbital and transcardially perfused with ice cold 0.9% saline. Brains were dissected and rapidly frozen in -80°C isopentane. Coronal brain sections were sliced using a cryostat (-20°C) at a thickness of 200 *μ*m. The peri-infarct (Bregma +1.0 to −1.0 mm), hippocampus (Bregma -1.2 to -2.5 mm), and thalamus (Bregma −1.2 to −2.2 mm) samples were obtained (Figure 1(a)) and stored frozen at -80°C until further analysis.

### 2.5. Protein Extraction and Western Blotting

**Figure 2:**
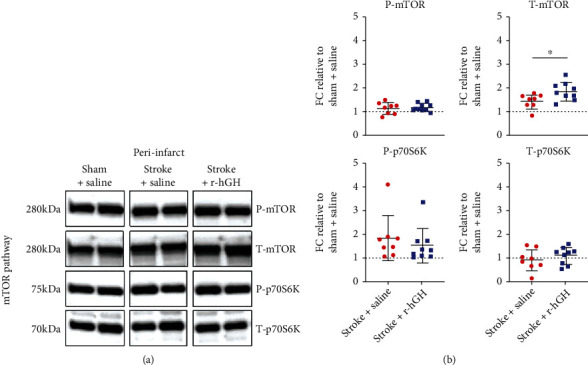
The effect of recombinant human growth factor (r-hGH) treatment poststroke on mammalian target of rapamycin (mTOR) in the peri-infarct region. (a) Representative blots of phosphorylated-mTOR (P-mTOR, active form), total-mTOR (T-mTOR), phosphorylated-p70S6-kinase P-p70S6K), and total-p70S6k (T-p70S6K). (b) T-mTOR and T-p70S6K levels were normalized to *β*-actin. P-mTOR and P-p70S6K levels were normalized to T-mTOR and T-p70S6K, respectively. Levels were expressed as a fold change (FC) of mean ± SD for each group relative to the mean of the Sham+Saline group (dotted line). We found a significant increase in T-mTOR protein expression within the peri-infarct region in stroked mice treated with r-hGH (blue squares) compared with saline (red circles). ∗*p* < 0.05, 2-tailed *t*-test.

**Figure 3:**
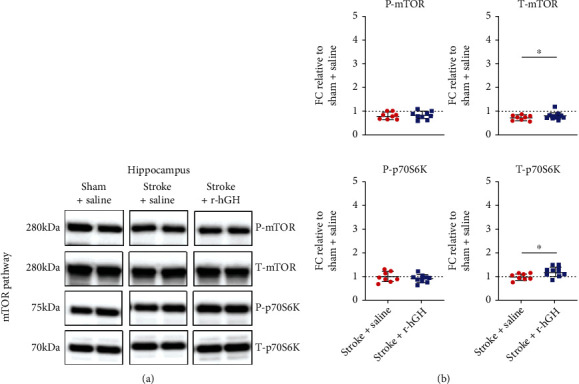
The effect of recombinant human growth factor (r-hGH) treatment poststroke on mammalian target of rapamycin (mTOR) in the hippocampus. (a) Representative blots of phosphorylated-mTOR (P-mTOR, active form), total-mTOR (T-mTOR), phosphorylated-p70s6-kinase (P-p70S6K), and total-p70s6k (T-p70S6K). (b) Data are presented as in Figure 2. We found a significant increase in T-p70S6K protein expression within the hippocampus in stroked mice treated with r-hGH (blue squares) compared with saline (red circles). ∗*p* < 0.05, 2-tailed *t*-test.

Protein extraction and western blotting were performed as previously described [7, 9, 10]. Tissue samples were sonicated in 300 *μ*l lysis buffer (50 mM TRIS buffer pH 7.4, 1 mM ethylenediaminetetraacetic acid, 1 mM dithiothreitol, 80 *μ*M ammoniummolybdate, 1 mM sodium pyrophosphate, 1 mM sodium vanadate, 5 mM *β*-glycerolphosphate, 1% SDS, 1 protease inhibitor cocktail tablet, 1 phosphatase inhibitor cocktail tablet, final concentration) and centrifuged at 14000*g* for 20 min at 4°C. Supernatants were collected, and protein concentrations were estimated by Pierce BCA protein assay kit (Thermo Fisher Scientific, USA) according to the manufacturer's instructions. Samples were mixed with sample buffer (2% SDS, 50 mM Tris, 10% glycerol, 1% DTT, 0.1% bromophenol blue, pH 6.8). 15 *μ*g of protein lysate was electrophoresed into Biorad Criterion TGX stain-free 4–20% gels and transferred to PVDF membranes. The membranes were blocked with 5% skim milk in TBST for 1 hour at room temperature and incubated overnight at 4°C with the appropriate primary antibody: BDNF, T-mTOR, P-mTOR, T-p70S6K, P-p70S6K, and *β*-actin (see Table 1 for antibody concentrations). The next day, membranes were incubated with the respective secondary antibody for 1 hour at 25°C. In between each incubation step, membranes were washed in TBST (3 × 10 min). Membranes were visualized on an Amersham Imager 600 using Luminata Classico western blotting detection reagent. The density of the bands was measured using Amersham Imager 600 analysis software. The densities corresponded linear to relative quantities, but these were given in arbitrary units. BDNF, T-mTOR, and T-p70S6K levels were normalized to *β*-actin. P-mTOR and P-p70S6K levels were normalized to T-mTOR and T-p70S6K, respectively. The data were expressed as a fold change of mean ± SD for each group relative to the mean of the Sham+Saline group. It should be noted that the blots were performed by an investigator who knew the treatment groups and the blots were also used to probe for multiple proteins. After imaging, blots were reprobed by washing in TBST (3 × 10 min) followed by incubation in Restore PLUS Western Blot Stripping Buffer (Thermo Scientific, USA) according to manufacturer's instructions and 1-hour incubation with blocking buffer (0.1% NaN_3_ with 5% BSA in TBST) followed by 3 × 10 min wash with TBST before reprobing with the next antibody. Raw blots can be viewed in Supplementary Material (Figures 1(a–f), 2(a–f), and 3(a–f)).

Note: The dataset of AMPA-receptor subunit GluR1 (GluR1, marker of synaptic plasticity), neuronal nuclei (NeuN, neuronal marker), and doublecortin (DCX, marker of neural migration) was an excerpt from previous studies [9, 10].

### 2.6. Statistical Analyses

In this exploratory investigation of r-hGH treatment [29], all data were presented as mean ± SD and were analyzed using GraphPad Prism v7.02. Data were analyzed using 2-tailed *t*-tests. Pearson correlation analysis was performed to assess relationships between expression levels of various proteins. The correlations were classified as tiny (*r* < 0.05), very small (0.05 < = *r* < 0.1), small (0.1 < = *r* < 0.2), medium (0.2 < = *r* < 0.3), large (0.3 < = *r* < 0.4), or very large (*r* > = 0.4) according to Funder and Ozer [30]. A *p* value <0.05 was considered statistically significant. The data that supports the findings for this study are available from the corresponding author upon reasonable request.

## 3. Results

### 3.1. GH Treatment Promotes pro-BDNF Expression

The antibody against BDNF labeled a protein band with a relative mw of about 32 kDa (Supplementary Figures 1A, 2A, 3A) which is consistent with the reported molecular weight of the uncleaved immature form of BDNF (pro-BDNF) [31].

The protein homogenates from the peri-infarct region of Stroke+Saline and Stroke+r-hGH were analyzed along with Sham+Saline using western blotting. We found significant increases in pro-BDNF levels across all regions in stroked mice treated with r-hGH compared with saline (peri-infarct, Stroke + Saline fold change (FC) = 1.24 ± 0.12, Stroke + r − hGH (FC) = 1.56 ± 0.19, *p* = 0.0011; hippocampus, Stroke + Saline FC = 0.83 ± 0.05, Stroke + r − hGH FC = 1.08 ± 0.19, *p* = 0.0033; and thalamus, Stroke + Saline FC = 1.13 ± 0.15, Stroke + r − hGH FC = 1.53 ± 0.34, *p* = 0.0076) (Figure 1(b), Table 2).

### 3.2. r-hGH Promotes Changes in the mTOR Signalling Pathway

In the peri-infarct area, r-hGH treatment significantly increased expression levels of T-mTOR (Stroke + Saline FC = 1.42 ± 0.3, Stroke + r − hGH FC = 1.83 ± 0.4, *p* = 0.0305) (Figure 2, Table 2). We did not observe any significant differences in the rest of the markers (P-mTOR, Stroke + Saline FC = 1.13 ± 0.25, Stroke + r − hGH FC = 1.18 ± 0.17, *p* = 0.7; T-p70S6K, Stroke + Saline FC = 0.91 ± 0.45, Stroke + r − hGH FC = 1.1 ± 0.35, *p* = 0.3; and P-p70S6K, Stroke + Saline FC = 1.8 ± 0.96, Stroke + r − hGH FC = 1.5 ± 0.74, *p* = 0.5).

In the hippocampus, treatment with r-hGH significantly increased protein levels of T-p70S6K (Stroke + Saline FC = 0.96 ± 0.14, Stroke + r − hGH FC = 1.2 ± 0.2, *p* = 0.0282) compared with saline (Figure 3, Table 2). We did not observe any significant differences in the rest of the markers (T-mTOR, Stroke + Saline FC = 0.74 ± 0.11, Stroke + r − hGH FC = 0.84 ± 0.15, *p* = 0.2; P-mTOR, Stroke + Saline FC = 0.8 ± 0.14, Stroke + r − hGH FC = 0.82 ± 0.17, *p* = 0.8; and P-p70S6K, Stroke + Saline FC = 0.97 ± 0.21, Stroke + r − hGH FC = 0.88 ± 0.18, *p* = 0.3).

**Figure 4:**
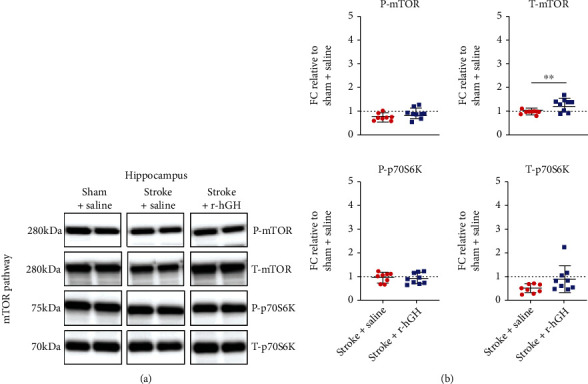
The effect of recombinant human growth factor (r-hGH) treatment poststroke on mammalian target of rapamycin (mTOR) in the thalamus. (a) Representative blots of phosphorylated-mTOR (P-mTOR, active form), total-mTOR (T-mTOR), phosphorylated-p70S6-kinase (P-p70S6K), and total-p70S6K (T-p70S6K). (b) Data are presented as in Figure 2. We found a significant increase in T-mTOR protein expression within the thalamus in stroked mice treated with r-hGH (blue squares) compared with saline (red circles). ∗∗*p* < 0.01, 2-tailed *t*-test.

Finally, in the thalamus r-hGH treatment poststroke significantly increased T-mTOR (Stroke + Saline FC = 0.96 ± 0.1, Stroke + r − hGH FC = 1.3 ± 0.27, *p* = 0.0093), (Figure 4, Table 2). There were no significant differences in the rest of the markers (P-mTOR, Stroke + Saline FC = 0.74 ± 0.16, r − hGH FC = 0.89 ± 0.23, *p* = 0.1; T-p70S6K, Saline FC = 0.5 ± 0.19, r − hGH FC = 0.92 ± 0.57, *p* = 0.06; and P-p70S6K, Saline FC = 0.99 ± 0.2, r − hGH FC = 0.94 ± 0.24, *p* = 0.7).

### 3.3. Pro-BDNF Expression Correlates with T-mTOR and Markers of Neurogenesis

#### 3.3.1. T-mTOR

**Figure 5:**
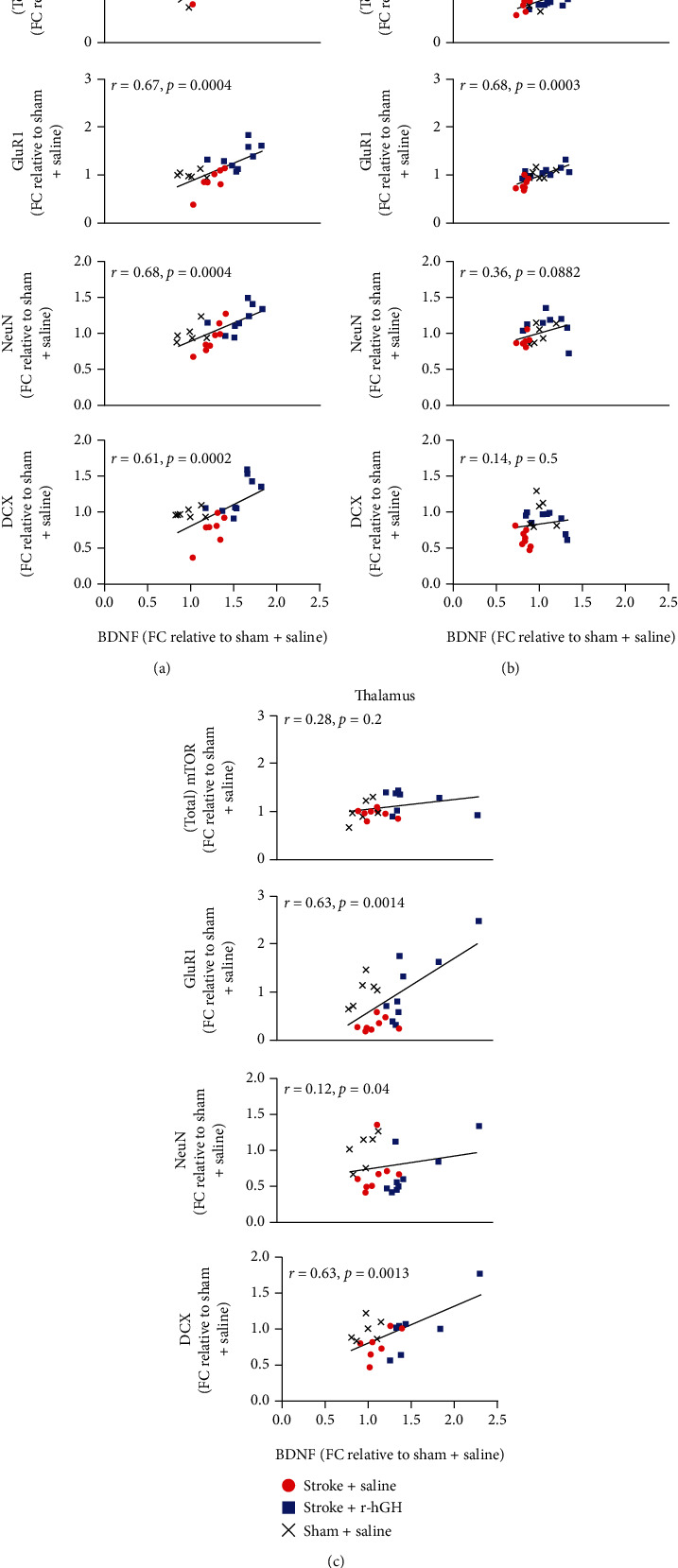
Correlation of uncleaved immature form of BDNF (pro-BDNF) with mammalian target of rapamycin (mTOR) and markers of neuroplasticity. Correlation of pro-BDNF, with T-mTOR (top panel), *α*-amino-3-hydroxy-5-methyl-4-isoxazolepropionic acid (AMPA)-receptor subunit GluR1 (marker of synaptic plasticity, second from top panel), NeuN (neuronal marker, third from top panel), and doublecortin (DCX, neuronal migration marker, bottom panel) in the peri-infarct region (a), hippocampus (b), and thalamus (c). Dataset GluR1, NeuN, and DCX was an excerpt from previous study [9, 28]. Correlations contain all three experimental groups, Sham+Saline (black crosses), Stroke+Saline (red circles), and Stroke+r-hGH (blue squares) and analyzed using Pearson's correlation. Pearson's *r* and associated *p* values for each correlation are reported on the corresponding graph.

There was a very large, significant correlation between pro-BDNF and T-mTOR in both the peri-infarct area (*r* = 0.86, *p* < 0.0001) and hippocampus (*r* = 0.54, *p* = 0.0076). There was no correlation observed in the thalamus (*r* = 0.28, *p* = 0.2) (Figure 5, Table 2).

### 3.4. GluR1

There was a very large, significant correlation between pro-BDNF and GluR1 across all locations (peri-infarct, *r* = 0.67, *p* = 0.0004; hippocampus, *r* = 0.68, *p* = 0.0003; thalamus, *r* = 0.63, *p* = 0.0014) (Figure 5, Table 2).

### 3.5. NeuN

We observed a very large correlation between pro-BDNF and NeuN within the peri-infarct area (*r* = 0.68, *p* = 0.0004). There were no significant correlations observed in either of the other investigated regions (hippocampus, *r* = 0.36, *p* = 0.0882; thalamus, *r* = 0.12, *p* = 0.4) (Figure 5, Table 2).

### 3.6. DCX

There was a significant and very large correlation between pro-BDNF and DCX observed in both the peri-infarct area and thalamus (*r* = 0.61, *p* = 0.002, and *r* = 0.63, *p* = 0.0013, respectively). There was no significant correlation observed in the hippocampus (*r* = 0.14, *p* = 0.5) (Figure 5, Table 2).

## 4. Conclusion

We have previously shown that r-hGH treatment after experimental stroke promotes neurorestorative processes in the peri-infarct area and hippocampus, leading to improvement in motor and cognitive functions [7, 9, 10]. In the current study, we extended upon these findings to investigate whether treatment with GH alters pro-BDNF and mTOR levels in the previously investigated peri-infarct area and the hippocampus, as well as the thalamus, which we have shown undergoes secondary neurodegeneration around 14 days poststroke [18, 20]. We found that r-hGH treatment after stroke resulted in a significant global increase of 25-35% in pro-BDNF expression in the investigated regions. Despite the global increase in pro-BDNF expression, the upregulation of mTOR protein was specific to the peri-infarct area and the thalamus. While a strong correlation may be suggestive of causal relationships or pathways, we would like to acknowledge that other associative relations may also be possible.

Nevertheless, significant correlations of pro-BDNF and mTOR protein expression, as well as markers of neuroplasticity, were confined to only the peri-infarct region. This suggests that other known signalling pathways upstream and/or downstream of BDNF or BDNF independent pathways may be activated to induce or limit neuroplasticity in the hippocampus and thalamus. For example, although an increase in the amount of pro-BDNF would suggest an increase in available substrate for BDNF to induce neuroplasticity, the cleaved (pro) portion of pro-BDNF and pro-BDNF itself can interact with p75 to stimulate mTOR. This would modulate neuroplasticity in the opposite direction to that of BDNF acting through trkB that could reduce or cancel out neuroplasticity in particular brain regions [32, 33]. Identification of these brain region-specific pathways may provide a way to develop targeted therapies to further enhance neuroplasticity and poststroke recovery.

r-hGH treatment at 2 days poststroke for 28 days increased the expression of pro-BDNF in the peri-infarct area, hippocampus, and thalamus. This is in agreement with a previous study by Zhang et al. [11] that showed 2 weeks of GH treatment commencing 8 weeks after TBI significantly increased brain BDNF levels in the hippocampus and prefrontal lobe. Zhang et al. [11] also showed that increased BDNF following GH treatment was associated with improved cognitive function, a finding that we have also reported in our previous publications [7, 9, 28]. BDNF has been shown to protect dopaminergic neurons that have been exposed to 1-methyl-4-phenyl-1,2,3,6-tetrahydropyrisine lesions [34] and serotonergic neurons that have been exposed to p-chloroamphetamine [35] and can protect cortical neurons from hypoxia-ischemia [36]. Potential mechanisms for the neuroprotective and neurorestorative effects of BDNF have been attributed to its binding to TrkB receptors, leading to reduced excitotoxicity, reduced free radical production, and the regeneration of damaged neurons and synaptic plasticity [37]. While we documented increased pro-BDNF levels across all regions, the upregulation of mTOR and markers of neuroplasticity is region-specific. Given the delayed nature of commencing GH treatment in our study (2 days poststroke), when cell death at the primary site of the occlusion is most likely complete, our current results suggest that at least some of the beneficial effects of GH treatment are likely to be through promoting pro-BDNF and neuroplasticity.

The predictive value of serum BDNF for stroke outcome is controversial, with some studies reporting that low BDNF is associated with poor outcome poststroke [38, 39], while others reported no or a weak association [38]. This may be explained by BDNF having very limited ability to cross the blood-brain barrier (BBB) [40]; therefore, peripheral levels of BDNF presumably poorly reflect brain levels [28]. Likewise, part of brain BDNF could be derived partly from the platelets within the circulation [41], which, with an injured BBB poststroke, could be of significant quantity. In our current study, with an injury that might compromise the BBB integrity, the source of increased BDNF in the brain is therefore not known. In addition to differences in BDNF origin with respect to BBB crossing, GH and IGF-I may also act by several pathways. Firstly, GH may have direct effects on the brain by crossing over the BBB and acts on GH receptors in the brain [42]. Secondly, GH treatment can increase IGF-1 [43], which can cross the BBB [44], and has been shown to increase BDNF expression within the hippocampus [45]. We have shown in our previous study that our GH treatment paradigm significantly increases both serum and brain IGF [7]. Interestingly, higher circulatory levels of IGF-1 correlate with better long-term outcome in stroke patients [46]. Our current finding suggests that the beneficial effects of GH treatment may be synergistically mediated by IGF-1 and pro-BDNF. Intravenous delivery of BDNF has been shown to improve motor outcome poststroke in preclinical studies [47]. However, difficulties of delivery and BBB permeability have limited its potential as a therapy to enhance poststroke functional outcomes and neuroplasticity. Our findings show that treatment with r-hGH can increase the expression of BDNF, therefore bypassing current difficulties of therapeutic delivery.

r-hGH treatment significantly increased T-mTOR protein expression, and pro-BDNF was significantly correlated with T-mTOR protein, GluR1, NeuN, and DCX within the peri-infarct region. This is in line with previous publications showing that BDNF-induced mTOR activation via the TrkB receptor induces neurogenesis [48, 49] and synaptic plasticity [50]. The beneficial effect of activating mTOR in the peri-infarct region in our study is in contrast with the previous literature showing that inhibition of mTOR with the prototypic mTOR inhibitor rapamycin is neuroprotective [16, 51]. This discrepancy may be explained by the timing of intervention. Previous studies of rapamycin administered drug just before or within 6 hours of stroke onset [16], when ischemia is actively ongoing, a scenario where a reduction in cellular metabolism and reduced GluR1 expression may be beneficial [15]. We commenced GH treatment at 48 hours after stroke and found that mTOR protein expression and GluR1 were increased after 28 days of r-hGH treatment, when cell death is most likely complete [52], indicating that increased mTOR and GluR1 expression during the recovery phase of stroke may be beneficial by increasing neuroplasticity and reducing tissue loss. Alternatively, hyperactivation of mTOR and increased GluR1 expression may represent a physiological reaction to the acute stroke insult. Indeed, further experiments where mTOR is inhibited and or GluR1 is expressed in the recovery phase of stroke are needed to test this hypothesis.

We found an increase in T-mTOR protein without an increase in P-mTOR relative to total protein expression. Insulin-mediated signalling through AKT has been shown to acutely increase mTOR activity by phosphorylation of Ser2448 on the mTOR catalytic domain [53]. Chronic interventions such as seven weeks of endurance and resistance exercise have been shown to increase expression of AKT and mTOR in skeletal muscle, indicating an increase in mTOR activity and ability to translate proteins for muscle growth [54]. The lack of change in P-mTOR in our study is likely due to a proportional increase in phosphorylation with increased protein expression and is likely indicative of a net increase in mTOR activity. However, we did not observe an increase in expression of T-p70S6K or protein phosphorylation. This may be due to mTOR signalling through its other downstream target, eukaryotic initiation factor 4E (eIF4E)-binding protein 1 (4EBP1), to induce cell proliferation and synaptic protein translation needed for neuroplasticity [55]. However, this interpretation would require further confirmatory studies.

r-hGH treatment did not alter levels of mTOR protein in the hippocampus. However, there was still a significant positive correlation between pro-BDNF levels and T-mTOR protein and GluR1 expression. This is, however, in contrast with a previous study showing that BDNF-induced mTOR signalling is required for GluR1 expression in the cornu ammonis 1 (CA1) subfield of the hippocampus, for consolidation of inhibitory avoidance in long-term memory formation [56]. We believe there may be three possible explanations for the divergence between r-hGH treatment, BDNF, and mTOR expression in the hippocampus. First, it may be due to a limitation in our tissue sampling. Western blotting analysis was carried out on the entire ipsilateral hippocampus; meaning, we were unable to differentiate changes in mTOR expression in the hippocampal subregions [57]. Furthermore, it has been shown that TrkB receptor gene expression varies between hippocampal subregions [58] and that the response of mTOR to acute brain ischemia differs in hippocampal subfields, with endogenous downregulation of mTOR activity occurring in the CA3 but not the CA1 subfield [59]. By sampling the whole ipsilateral hippocampus, we may have missed subtle alterations in these subregions, and opposing responses in certain subregions may have cancelled each other out. Additional immunohistochemical analysis of hippocampal subregions will help shed light on regional specific alterations in mTOR signalling in response to GH treatment. Second, BDNF-TrkB has also been shown to signal through the phospholipase C*γ* (PLC*γ*) and the mitogen-activated protein kinase (MAPK) pathways [14]. The PLC*γ* pathway can induce calcium transients leading to increased translocation of GluR1 subunits to synapsis in cultured cortical pyramidal neurons [60]. Finally, the MAPK signalling has been shown to increase transcription via 4EPB1 and S6 ribosomal proteins during synaptic plasticity [61]. This may explain why in our study r-hGH-treated animals had increased expression of p70S6K protein, independent of increases in mTOR protein expression. Taken together these results suggest that BDNF may signal through an mTOR-independent pathway to induce neuroplasticity and improve cognitive function poststroke. Our study was not able to discern between these possibilities.

Our previous studies have shown that secondary neurodegeneration becomes apparent in the thalamus around 14 days poststroke, specifically in the posterior complex and ventral posterolateral nucleus which are connected to the sensory and motor cortices (which was also the primary target of the photothrombotic stroke) [18–20]. Therefore, we extended our investigation to determine whether GH treatment elicited neurorestorative processes in the thalamus. We found that GH treatment significantly increased pro-BDNF and T-mTOR protein within thalamus. Pro-BDNF was only correlated with GluR1 receptor expression. These results suggest that GH treatment induces some neurorestorative processes within the thalamus, such as increasing pro-BDNF and T-mTOR protein levels and a significant correlation with pro-BDNF levels.

It should be noted that our study has some limitations. This is a cross-sectional study at 30 days poststroke (following 28 days of GH treatment). While we have identified several correlations of pro-BDNF, mTOR, and markers of neuroplasticity, we would like to acknowledge that the association may not be causal relationships. The usage of TrkB inhibitor or rapamycin could be used to determine whether the effects of GH treatment are mediated by BDNF and mTOR pathways. Furthermore, we only looked at changes in the ipsilateral regions in the current study; meaning, we may have missed important treatment-induced changes in the contralateral brain regions. This warrants further investigation in future studies. Finally, we commenced infusion of r-hGH at 2 days poststroke which continued for the remaining 28-day recovery period. Previous studies suggest that there may be a critical window starting at 14 days post-injury where regeneration is much more difficult owing to the upregulation of factors opposing regeneration (e.g., NOGO-A) [24, 62]. A key future experiment would be to delay r-hGH infusion until 14 days to determine whether GH is able to counteract the expression of the factors opposing to regeneration and to improve functional recovery during this critical period of time.

In conclusion, the present study reports novel evidence that although GH treatment increases pro-BDNF in multiple brain regions associated with motor and cognitive function poststroke, the neurorestorative actions of BDNF and the role of mTOR in these actions appear to be brain region-specific and mostly confined to the peri-infarct area. Collectively, our findings provide important insights into complex and brain region-specific mechanisms of action of GH-induced improvements in cognitive and motor function following stroke. Future studies in this space may open new avenues of investigation into pharmacologically enhancing brain recovery through signalling pathways such as mTOR downstream of BDNF.

## Figures and Tables

**Table 1:** List of antibodies used for western blot analyses.

	Sources of antibodies	Dilution
BDNF	Santa Cruz, polyclonal rabbit anti-BDNF (precursor and mature), sc-546	1 : 5000
T-mTOR	Cell Signalling, monoclonal mouse anti-total- mTOR, #4517	1 : 1000
P-mTOR	Cell Signalling, monoclonal rabbit anti-phospho-mTOR (Ser2448), #5536	1 : 500
T-p70S6K	Cell Signalling, polyclonal rabbit anti-total-p70S6K, #9202	1 : 1000
P-p70S6K	Cell Signalling, monoclonal mouse anti-phospho-p70S6K, #9206	1 : 2000
*β*-actin	Sigma-Aldrich, monoclonal anti-*β*-actin-HRP antibody, A3854	1 : 50000
Rabbit IgG	Biorad, anti-rabbit-HRP antibody, #170-6515	1 : 7500
Mouse IgG	Biorad, anti-mouse-HRP antibody, #170-6516	1 : 10000

**Table 2:** *t*-test and Pearson's results summary table.

*t*-tests						
Peri-infarct		Pro-BDNF	T-mTOR	P-mTOR	T-p70S6K	P-p70S6K
	FC ± SD (Stoke+Saline vs. Stroke+r-hGH)	1.24 ± 0.12 vs. 1.56 ± 0.19	1.42 ± 0.3 vs. 1.83 ± 0.4	FC = 1.13 ± 0.25 vs. 1.18 ± 0.17	0.91 ± 0.45 vs. 1.1 ± 0.35	1.8 ± 0.96 vs. 1.5 ± 0.74
	T, df, *p* value	4.029, 15, 0.0011	2.389, 5, 0.0305	0.4491,15, 0.7	0.9922, 15, 0.3	0.7802, 15, 0.5
Hippocampus		Pro-BDNF	T-mTOR	P-mTOR	T-p70S6K	P-p70S6K
	FC ± SD (Stoke+Saline vs. Stroke+r-hGH)	0.83 ± 0.05 vs. 1.08 ± 0.19	0.74 ± 0.11 vs. 0.84 ± 0.15	0.8 ± 0.14 vs. 0.82 ± 0.17	0.96 ± 0.14 vs. 1.2 ± 0.2	0.97 ± 0.21 vs. 0.88 ± 0.18
	T, df, *p* value	3.487, 15, 0.0033	1.431, 15, 0.2	0.3006, 15, 0.8	2.429, 15, 0.0282	0.9843, 15, 0.3
Thalamus		Pro-BDNF	T-mTOR	P-mTOR	T-p70S6K	P-p70S6K
	FC ± SD (Stoke+Saline vs. Stroke+r-hGH)	1.13 ± 0.15 vs. 1.53 ± 0.34	0.96 ± 0.1 vs. 1.3 ± 0.27	0.74 ± 0.16 vs. 0.89 ± 0.23	0.5 ± 0.19 vs. 0.92 ± 0.57	0.99 ± 0.2 vs. 0.94 ± 0.24
	T, df, *p* value	3.082, 15, *p* = 0.0076	2.984, 15, *p* = 0.0093	1.598, 15, *p* = 0.1	2.030, 15, *p* = 0.06	0.4560, 15, *p* = 0.7
Pearson's correlation						
Peri-infarct		Pro-BDNF and T-mTOR	Pro-BDNF and GluR1	Pro-BDNF and NeuN	Pro-BDNF and DCX	
	Pearson's *r*	0.86	0.67	0.68	0.61	
	*p* value	<0.0001	0.0004	0.0004	0.002	
Hippocampus		Pro-BDNF and t-mTOR	Pro-BDNF and GluR1	Pro-BDNF and NeuN	Pro-BDNF and DCX	
	Pearson's *r*	0.54	0.68	0.36	0.14	
	*p* value	0.0076	0.0003	0.0882	0.5	
Thalamus		Pro-BDNF and t-mTOR	Pro-BDNF and GluR1	Pro-BDNF and NeuN	Pro-BDNF and DCX	
	Pearson's *r*	0.28	0.63	0.12	0.63	
	*p* value	0.2	0.0014	0.4	0.0013	

FC = fold change; SD = standard deviation; *r* = rho.

## Data Availability

The data that support the findings of this study are available from the corresponding author upon reasonable request.

## Supplementary Materials

Supplementary Materials Supplementary Material File 1 contains the raw western blot images and the graphical abstract for this paper.
