# Supplementary material for: Growth Hormone Increases BDNF and mTOR Expression in Specific Brain Regions after Photothrombotic Stroke in Mice
Source: Neural Plast. 2022 Apr 15;2022:9983042. doi: 10.1155/2022/9983042 (PMC9033347; doi:10.1155/2022/9983042)
Supplement: Supplementary Materials — Supplementary Material File 1 contains the raw western blot images and the graphical abstract for this paper. [file 9983042.f1.docx]

**SUPPLEMENTARY MATERIAL B – Raw Western Blots**

**Investigating growth hormone treatment induced changes to BDNF and mTOR expression after photothrombotic stroke in mice**

Sonia Sanchez-Bezanilla^*^, Daniel J Beard^*^, Rebecca J Hood^*^, N. David Åberg, Patricia Crock, Frederick R. Walker, Michael Nilsson, Jörgen Isgaard†, Lin Kooi Ong†

**Western blot images – Peri-infarct**

1. **Full unedited blots for Figure 1B and 2 analysis – used in the analysis of A) BDNF, B) T-mTOR, C) P-mTOR, D) T-p70s6k, E) P-p70s6k and F) β-actin loading control western blots (peri-infarct samples).** The panels are images taken using an Amersham Imager 600 for bright field and chemiluminescent overlay, which provides information of the molecular weight/size of the bands (weights depicted to left of blots). The red boxes indicate the bands featured in Figure 1B and the corresponding rows in the remaining blots feature in Figure 2. The signals of the bands from the original, unprocessed immunoblots were measured using Amersham Imager 600 Analysis Software.

**Western blot images – Hippocampus**

1. **Full unedited blots for Figure 1B and 3 analysis – used in the analysis of A) BDNF, B) T-mTOR, C) P-mTOR, D) T-p70s6k, E) P-p70s6k and F) β-actin loading control western blots (hippocampus samples).** The panels are images taken using an Amersham Imager 600 for bright field and chemiluminescent overlay, which provides information of the molecular weight/size of the bands (weights depicted to left of blots). The red boxes indicate the bands featured in Figure 1B and the corresponding rows in the remaining blots feature in Figure 3. The signals of the bands from the original, unprocessed immunoblots were measured using Amersham Imager 600 Analysis Software.

**Western blot images – Thalamus**

1. **Full unedited blots for the Figure 1B and 4 analysis – used in the analysis of A) BDNF, B) T-mTOR, C) P-mTOR, D) T-p70s6k, E) P-p70s6k and F) β-actin loading control western blots (thalamic samples).** The panels are images taken using an Amersham Imager 600 for bright field and chemiluminescent overlay, which provides information of the molecular weight/size of the bands (weights depicted to left of blots). The red boxes indicate the bands featured in Figure 1B and the corresponding rows in the remaining blots feature in Figure 4. The signals of the bands from the original, unprocessed immunoblots were measured using Amersham Imager 600 Analysis Software.

Graphical Abstract


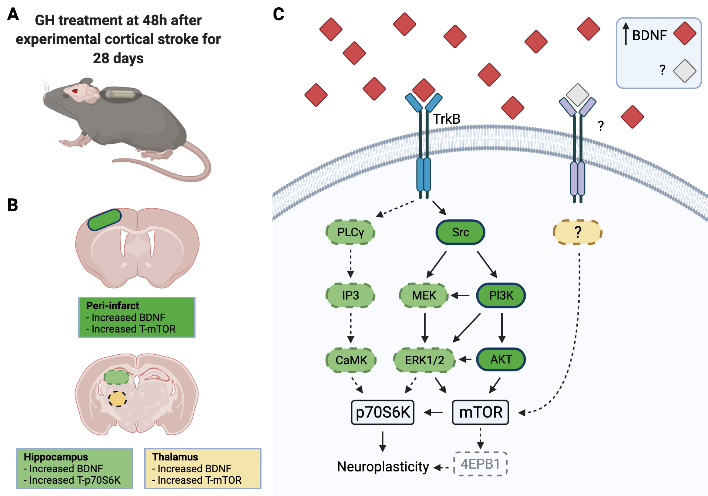


Growth hormone treatment after experimental stroke **(A),** increased brain derived neurotrophic factor (BDNF) expression in the peri-infarct area, thalamus and hippocampus **(B)**. BDNF expression was associated with mammalian target of rapamycin (mTOR) protein and markers of neuroplasticity in peri-infarct area, suggesting at least some of the beneficial effects of GH treatment are likely to be through promoting BDNF and neuroplasticity. The role of mTOR in these actions appear to be brain region specific **(C).**
